# Adapting Interventions for Home Hospice Caregivers Using Digital Health Innovation

**DOI:** 10.2196/81589

**Published:** 2026-01-14

**Authors:** Qing Huang

**Affiliations:** 1 Department of Research & Data Analytics Singapore Cancer Society Singapore Singapore

**Keywords:** palliative, hospice, home care, cancer, meta-synthesis

The recent meta-synthesis by Deng et al [1] on the experiences of family caregivers in home-based cancer hospice care was read with great interest. The authors presented a precise summary of their needs and perceptions and, in doing so, highlighted a critical gap in tailored interventions for supporting this vulnerable group. This is a gap that warrants further consideration.

This gap, while concerning, is understandable. The time and physical confinement inherent in home hospice care limit caregivers’ ability to participate in research or access traditional in-person support. Deng et al’s [1] review emphasized the dual challenges of this role: the immense burden of being “physically and emotionally present” and the critical need for “sharing responsibility” with palliative care teams, community partners, and family. While this shared support makes caregivers feel less isolated, the home hospice care setting creates logistical barriers to effective collaboration. To make this sharing of responsibility more accessible for home hospice caregivers, we should leverage innovative technology to connect the caregivers with their external support network. This is where we can adopt proven therapies like mindfulness-based stress reduction, delivering them through flexible digital methods that fit the specific constraints of the home hospice setting.

Encouragingly, some recent studies have validated this approach. For example, a pilot study using virtual reality demonstrated that technology can provide homebound caregivers with much-needed individual psychological respite through immersive nature scenes [2]. On a larger scale, a randomized controlled trial of the “Symptom Care at Home” intervention proved that an automated digital coaching system could significantly reduce caregiver burden by connecting them to their care team when symptoms escalated [3]. These studies illustrate the potential of novel ways of delivering support to overcome the practical barriers of home hospice care.

The work of Deng et al [1] should be a catalyst for action. To translate these promising findings into real-world practice, robust collaboration is essential for adapting and testing such interventions. An ideal model would involve researchers and developers co-designing digital tools with caregivers, an approach shown to improve the usability and adoption of digital health interventions [4]. This also calls for a partnership between hospice providers and community organizations to bring these tools into routine care, creating hybrid support models that combine scalable technology for both individual coping and shared care. Through such collaborative efforts, we can deliver interventions that truly empower caregivers to navigate one of life’s most profound challenges.
